# The Translational Benefits of Sheep as Large Animal Models of Human Neurological Disorders

**DOI:** 10.3389/fvets.2022.831838

**Published:** 2022-02-15

**Authors:** Samantha J. Murray, Nadia L. Mitchell

**Affiliations:** Faculty of Agriculture and Life Sciences, Lincoln University, Canterbury, New Zealand

**Keywords:** sheep, neuroscience, genetics, neurodegeneration, traumatic brain injury, stroke, epilepsy, spinal cord injury

## Abstract

The past two decades have seen a considerable rise in the use of sheep to model human neurological disorders. While each animal model has its merits, sheep have many advantages over small animal models when it comes to studies on the brain. In particular, sheep have brains more comparable in size and structure to the human brain. They also have much longer life spans and are docile animals, making them useful for a wide range of *in vivo* studies. Sheep are amenable to regular blood and cerebrospinal fluid sampling which aids in biomarker discovery and monitoring of treatment efficacy. Several neurological diseases have been found to occur naturally in sheep, however sheep can also be genetically engineered or experimentally manipulated to recapitulate disease or injury. Many of these types of sheep models are currently being used for pre-clinical therapeutic trials, particularly gene therapy, with studies from several models culminating in potential treatments moving into clinical trials. This review will provide an overview of the benefits of using sheep to model neurological conditions, and highlight naturally occurring and experimentally induced sheep models that have demonstrated translational validity.

## Introduction

Over the past two decades, the use of sheep (*Ovis aries*) to model human neurological disorders has become increasingly popular. Whilst the knowledge scientists have gained from the study of small animal models of neurological disorders is invaluable, sheep possess many advantages over smaller animals in studies concerned with modeling conditions of the human brain. In particular, studies of neurological diseases in sheep have been enhanced in recent years with the production of a fully annotated sheep genome, as well as detailed 3D magnetic resonance image (MRI) atlases and the Michigan State University histological coronal atlas of the sheep brain (1–8).

**Figure 1:**
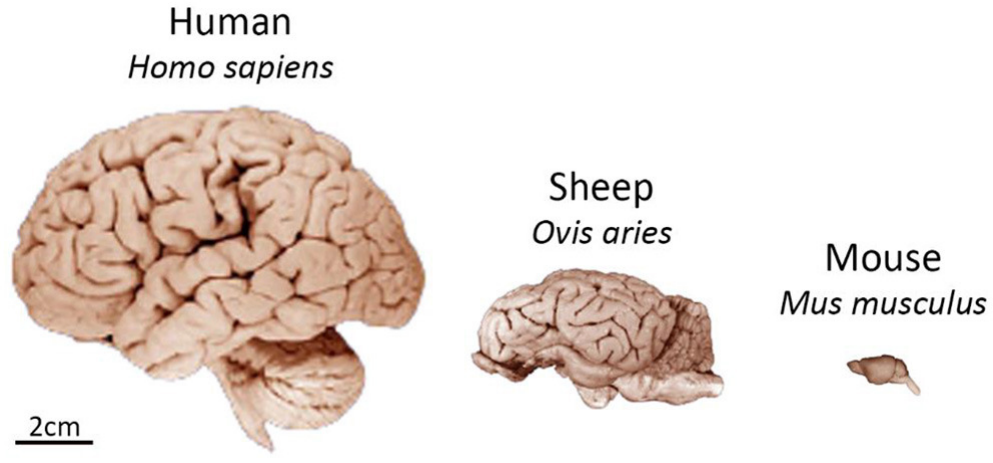
Comparison of size and gross neuroanatomy of the sheep, human, and mouse brain. Images reproduced from the University of Wisconsin and Michigan State Comparative Mammalian Brain Collections and National Museum of Health and Medicine. Preparation of images and specimens funded by the National Science Foundation, as well as by the National Institutes of Health. University of Wisconsin and Michigan State Comparative Mammalian Brain Collections website http://neurosciencelibrary.org/index.html.

First and foremost, sheep have a brain more comparable in size and anatomy to the human brain than smaller commonly used laboratory animals. The average weight of an adult sheep brain is 130–140 g, compared to 1–2 g for a rodent brain and 1,300–1,400 g for an adult human brain [Figure 1, (9)]. With that, the sheep brain shares a high level of structural homology to the human brain. Sheep have a gyrencephalic cortex and well defined basal ganglia nuclei, particularly the caudate nucleus and putamen separated by the internal capsule. There are also similarities in spine length, spinal canal width, and cerebrospinal fluid (CSF) volume between humans and sheep (10–12). Sheep have neurogenic niches in the dentate gyrus and sub-ventricular zone (SVZ), with a neuronal migratory stream from the SVZ to the olfactory bulb, as has been observed in the human brain (13). In addition, the morphology of the neuromuscular junction of sheep has been shown to closely resemble that of humans (14).

Sheep have a long life span relative to other research animals, with a natural life expectancy of 9–12 years. This is particularly useful when studying slow, progressive spontaneous diseases like neurodegenerative disorders as it allows the disease to progress at a more natural rate compared to the often-accelerated forms of disease in small animal models. Sheep are also similarly sized to humans, weighing 3.5–4.5 kg at birth and 80–110 kg as adults. This longer life span and similar body weight make sheep ideal for testing the dosage, distribution, and safety of pharmaceutical therapeutics. Normal healthy sheep can serve as large animal alternatives to non-human primates to trial new drug delivery techniques, compare the spread of different gene therapy viral vectors, or in pre-clinical safety and efficacy trials of drugs which have been successful in small animal models (16, 17). Increasingly, novel human therapeutics are being developed in diseased sheep models and receiving United States Food and Drug Administration (FDA) clearance for clinical translation (18–20).

Sheep are relatively cheap and easy to house, as in most cases they can continue to live in their natural environment in a typical farming situation. This is in stark contrast to small animal models, particularly rodents, which are housed in highly controlled environments. In addition, rodent breeding is also highly controlled and primarily involves inbreeding, leading to a relatively homogenous population (21–23). Sheep are more outbred, and this reflects in the heterogeneity of the population, which is more representative of the human population particularly when it comes to disease pathophysiology.

**Figure 2:**
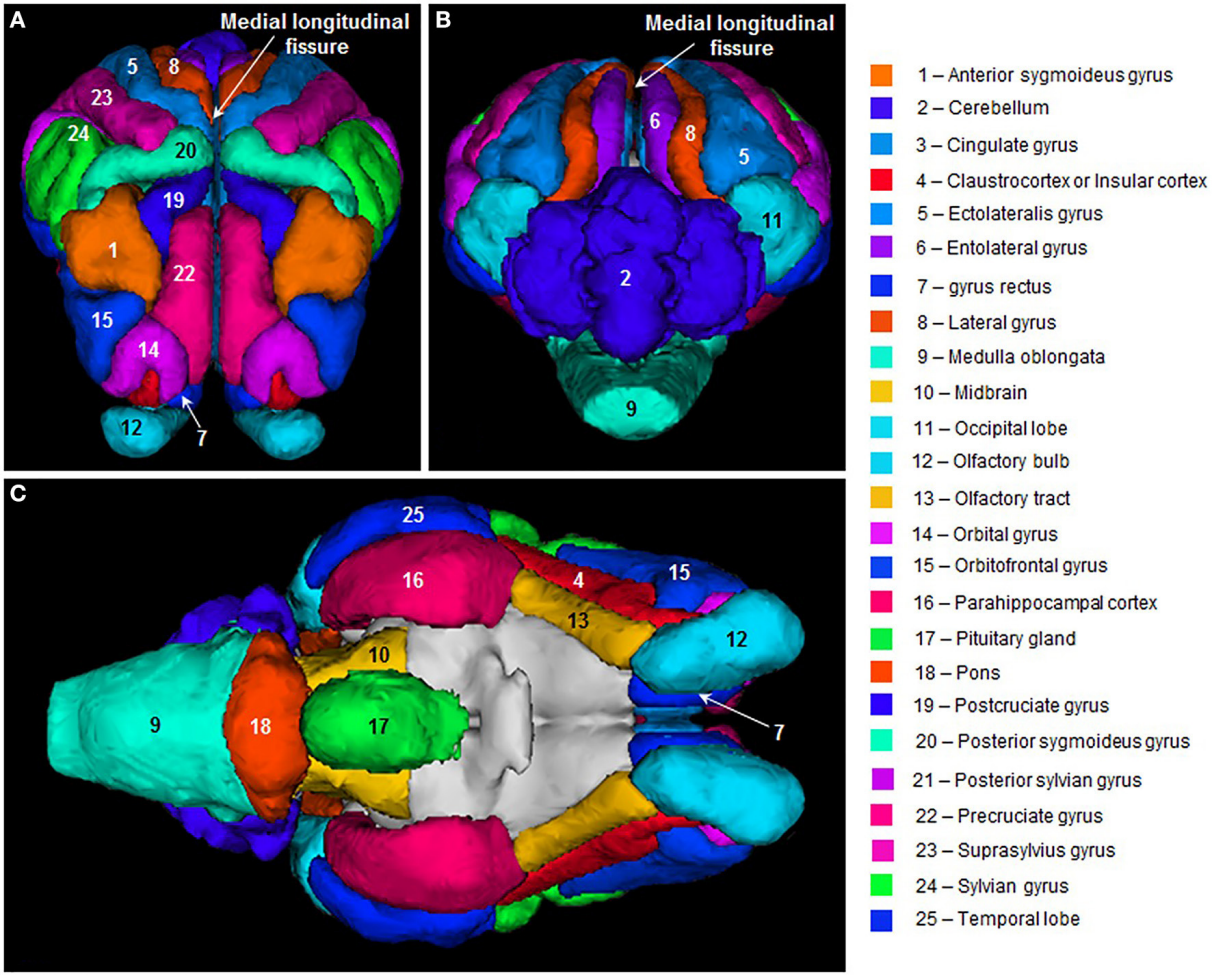
Sheep brain MRI atlas. 3D surface renderings of gyri segmented from a population-average template MR images of sheep brains displayed in: **(A)** frontal, **(B)** posterior, and **(C)** ventral views. Figure reproduced from *Computation of a high-resolution MRI 3D stereotaxic atlas of the sheep brain* by Ella et al. (1) with permission from John Wiley and Sons.

Being reasonably docile animals, sheep are easy to work with and can be assessed using a wide range of *in vivo* monitoring techniques including electroencephalography (EEG), electromyography (EMG), electroretinography, MRI (Figure 2), computed tomography, and actimetry (20, 24–30). Techniques such as EEG, EMG, and actimetry can be performed on awake, free-moving sheep, as they are strong and passive enough to carry a battery pack or transmitting device with them. Sheep are also intelligent animals and can be trained to perform cognitive tasks such as two-choice discrimination, facial recognition, reversal learning, and maze navigation (20, 31–34).

The Online Mendelian Inheritance in Animals (OMIA) database has 108 listings under potential models for human disease in sheep, of which 28 are neurological disorders or have a neurological component (https://omia.org/home/). Several neurodegenerative lysosomal storage diseases occur naturally in sheep, requiring no experimental intervention to produce a phenotype and associated neuropathology. These include models of Neuronal Ceroid Lipofuscinosis (NCL), Tay-Sachs disease, and Gaucher disease (35–38). Another neurodegenerative disease, Huntington's disease, is modeled in transgenic sheep expressing the human form of the mutated Huntingtin gene (39). More recently, CRISPR-Cas9 gene editing technology has been successfully employed to generate a sheep model of CLN1 NCL (40). Furthermore, non-genetic neurological disorders can be experimentally induced in sheep, through the use of instrumentation, mechanical force, or bacterial infection. Examples include traumatic brain injury, spinal cord injury, stroke, and epilepsy (12, 41–43). This review will provide an overview of the models presented above, with a particular focus on how the use of sheep to model these diseases is advancing pre-clinical research.

## Naturally Occurring Sheep Disease Models

### Neuronal Ceroid Lipofuscinosis (CLN5 and CLN6 Batten Disease)

Neuronal ceroid lipofuscinosis (NCL), also known as Batten disease, is an inherited neurodegenerative lysosomal storage disorder affecting predominantly infants and children. It is inherited primarily in an autosomal recessive pattern, and affects ~1 in 12,500 individuals worldwide (44). To date 13 different variants of NCL have been identified, they are classified by the affected gene and the age of onset (45, 46). Clinical symptoms include seizures, progressive loss of vision, and progressive motor and cognitive decline, although the onset and severity of these differs between disease variants. A hallmark pathological feature of NCLs is the accumulation of autofluorescent storage material in lysosomes. In addition, there is widespread atrophy throughout the brain and significant neuronal loss.

Many of the 13 forms of NCL also occur naturally in other mammals including non-human primates, dogs, cows, and sheep. The best characterized of these is a CLN6 form in New Zealand South Hampshire sheep, which has been under active study since the 1970's (47). Subsequently a second CLN6 disease model was identified in Australian Merino sheep (48), and both result from mutations in the *CLN6* gene (37). Naturally occurring CLN5 disease was discovered in New Zealand Borderdale sheep in the early 2000's and is due to a nucleotide substitution at a consensus splice site in the *CLN5* gene resulting in a truncated non-functioning CLN5 protein in affected sheep (35, 49). In humans, mutations in both CLN5 and CLN6 primarily result in late infantile onset NCL. The disease presentation in affected sheep is similar to that of humans, with progressive brain atrophy, cognitive decline, and loss of vision and these sheep have been instrumental in better understanding NCL pathophysiology and more recently the development of therapies.

Gene therapy to replace the defective NCL gene in affected South Hampshire (CLN6^−/−^) and Borderdale (CLN5^−/−^) sheep has produced encouraging results, particularly in CLN5^−/−^ sheep. Intracerebroventricular (ICV) administration of an adeno-associated virus serotype 9 (AAV9) carrying *CLN5* was able to prevent or halt brain atrophy and cognitive decline in both pre- and post-symptomatic CLN5^−/−^ sheep, and delay but not prevent loss of vision (20). To address vision loss directly, affected sheep received AAV9-mediated gene therapy *via* the vitreous humor (intravitreal; IVT) in a single eye, with the contralateral eye acting as an internal untreated control (50). In CLN5^−/−^ sheep a single dose of IVT gene therapy was able to attenuate retinal dysfunction and pathology in the treated eye. In September 2021, the CLN5 gene therapy product was cleared as a candidate investigational new drug by the FDA, based on yet-to-be published studies performed in CLN5-/- Borderdale sheep in New Zealand.

### Gaucher Disease

Gaucher disease is another lysosomal storage disorder that presents naturally in sheep. It is caused by mutations in the β-glucocerebrosidase (*GBA*) gene, resulting in deficiencies in the lysosomal enzyme glucocerebrosidase and consequently accumulation of lipids in cells, primarily macrophages, throughout the body (51). Gaucher disease is inherited in an autosomal recessive manner and has a prevalence of between 1 in 40,000 and 1 in 60,000 births. There have been over 300 disease-causing mutations found in the *GBA* gene, which have been broadly categorized into three subtypes of Gaucher disease (51, 52). Type I is a non-neuronopathic form presenting anywhere from childhood through to adulthood, with patients suffering from splenomegaly, hepatomegaly, and bone disease. Type II and III have similar visceral manifestations to Type I, but also include a neurological phenotype. Patients with neuronopathic Gaucher disease can suffer from progressive epilepsy, cerebellar ataxia, dementia, and psychomotor and oculomotor deficits (51, 52).

A naturally occurring model of Gaucher disease was identified in Australian Southdown sheep after lambs were born several years in a row with severe motor abnormalities, seizure activity, and thick leathery skin. These clinical signs were accompanied by extremely low glucocerebrosidase activity in the blood and cultured skin fibroblasts and high levels of its substrate, glucocerebroside, in the blood, liver, and brain (36). Genetic analysis revealed homozygous missense mutations in the ovine *GBA* gene, which in human patients leads to an acute neuronopathic, or Type II, phenotype. Sheep with neuronopathic Gaucher disease have been invaluable in studying lipid homeostasis and cellular membrane composition in neurodegenerative disease states in a large animal model (53, 54). However, sheep with Gaucher disease typically die within a few days of birth therefore establishing a research flock to study disease progression or assess long-term efficacy of potential therapeutics is not an option. Nevertheless, heterozygous mutations in *GBA* have recently been found to be a high genetic risk factor for Parkinson's disease [PD; (55)], therefore heterozygous carriers in these flocks have the potential to be developed into a large animal model to further elucidate the relationship between *GBA* and PD.

### Tay-Sachs Disease

Tay-Sachs disease is one of several GM2 gangliosidoses, another subset of lysosomal storage diseases. Tay-Sachs disease is characterized by reduced hexosaminidase A (Hex A) enzyme activity as a result of missense mutations in the *HEXA* gene. Loss of function of the α subunit of Hex A leads to the accumulation of gangliosides in the lysosomes of neurons (56). Patients therefore display a progressive neurological phenotype including weakness and hypotonia, ataxia, nystagmus, vision loss, and seizures. Disease incidence in the general population is reported to be 1 in 320,000, however there is a much higher rate of disease in people of Ashkenazi Jewish descent (57). Clinical onset of disease varies from infancy, where disease symptoms are severe and progress rapidly, to early adulthood where disease progression in much more gradual (56, 58). Key pathological characteristics include a macula “cherry-red spot,” atrophy of the cortex, brainstem, and cerebellum, neuroinflammation, and lysosomal storage in macrophages throughout the body (58).

Tay-Sachs disease has been described in North American and British Jacob sheep that have a single base substitution in exon 11 of the *HEXA* gene, resulting in skipping of exon 11 and diminished Hex A activity (38, 59). Jacob sheep are currently the only animal model of Tay-Sachs disease that displays measurable clinical symptoms and robust neuropathology. Sheep begin to show neurological symptoms such as gait abnormalities, proprioceptive deficits, vision loss, and tremors between 6 and 8 months of age (38, 59, 60). Tay-Sachs sheep have enlarged neuronal cell bodies throughout the brain and spinal cord due to the accumulation of gangliosides, astrogliosis, and microgliosis throughout the brain (60, 61).

Owing to their robust phenotype and disease-related pathology, Tay-Sachs sheep are the ideal model for testing potential therapies for GM2 gangliosidoses. Indeed, AAVrh8-mediated gene therapy trials in Tay-Sachs sheep demonstrated safety and efficacy, with widespread vector distribution throughout the brain, as well as near normal levels of Hex A activity and attenuated ganglioside accumulation (19). This resulted in delayed onset of disease and increased life span in treated sheep and the initiation of a phase I clinical trial of the HEXA gene therapy product in January 2021 [clinicaltrials.gov NCT04669535, (62)].

### Transmissible Spongiform Encephalopathy

Transmissible spongiform encephalopathies (TSEs) are a group of infectious neurodegenerative diseases, also known as prion diseases, including Creutzfeld-Jakob disease (CJD) in humans and scrapie in sheep. TSEs result from the accumulation of an abnormal protease resistant isoform of prion protein (PrP), known as a scrapie associated prion protein or PrP^Sc^.

In humans, CJD has an incidence of 1–2 per million worldwide and can be subclassified as familial, acquired (i.e., through medical procedures) or, the most common form, sporadic CJD (63, 64). There is also variant CJD which is primarily caused by the consumption of TSE-infected meat products. Clinical presentation of CJD is very heterogeneous however common symptoms include cognitive decline, myoclonus, motor deficits, and dementia (65). Pathologically CJD is characterized by neuronal cell loss, accumulation of abnormal prion protein (PrP^Sc^), and vacuolisation of cell bodies throughout the cerebral cortex and subcortical structures.

Scrapie in sheep was the first prion disease to be identified and was given its name due to the common clinical sign of sheep scraping their fleece off against fences, trees, or rocks. Scrapie can be defined as either classical or atypical, with atypical scrapie also known as Nor98-like scrapie after its discovery in Norway in 1998 (66). Clinical signs of classical scrapie include itching (pruritus) and scraping of fleece, ataxia, loss of body condition, and behavioral changes (67, 68). Atypical scrapie cases can often go undetected until post-mortem due to the lack of, or less severe, clinical signs. In particular, atypical scrapie lacks the fleece scraping and itching response but can present with ataxia, tremor and poor body condition (67, 69, 70). Post-mortem assessment of central nervous system tissues in cases of classical scrapie reveal neuronal cell loss, gliosis, accumulation of PrP^Sc^ and associated vacuolisation of neuronal cell bodies, with the brainstem being most severely affected (71–73). In contrast, these neuropathological features are less intense in the brainstem of atypical scrapie cases. PrP^Sc^ deposits and the resulting cellular vacuolisation is found throughout the cerebral cortices and cerebellum in atypical scrapie (66, 74).

Studying the genetics, proteomics, neuropathology, and infectivity of prion diseases in sheep has been a valuable tool in understanding the human prion diseases, particularly in studying the transmission of prion proteins through blood (75, 76). Houston and colleagues used sheep to demonstrate the ability of TSEs to be transmitted to a recipient through a blood transfusion (76). The finding that blood was infectious even when donor sheep were asymptomatic has important clinical implications (75, 76). Sheep made good models in this case as their similar body size to humans meant blood transfusion volumes were clinically relevant. In addition, because TSEs occur naturally in sheep, the infectivity of blood was able to be tested from naturally infected donors as well as experimentally infected donors (75). Sheep naturally infected with scrapie have also been used to validate an assay for detecting minute levels of prion protein in biological samples. Importantly, scaling this assay up from small animals to sheep, researchers were able to demonstrate the ability of the assay to also detect prion protein in CSF in a large animal, which has translated to the development of non-invasive diagnostic screening in humans (77–79). Sheep with naturally occurring scrapie are also being utilized to elucidate the relationship between prion proteins, neuroinflammation, and neurodegeneration, and to trial anti-inflammatory drugs such as glucocorticoids as potential treatments (80–82).

## Experimentally Induced Sheep Disease Models

### Genetically Engineered Models

#### Huntington's Disease

Huntington's disease (HD) is an inherited neurodegenerative disease, first described by George Huntington in 1872. The prevalence of Huntington's disease varies greatly between continents, but on average affects 5 to 10 in 100,000 individuals worldwide (83). Patients typically manifest with a triad of psychiatric, motor, and cognitive symptoms, with severity and age of onset differing widely between individuals, and death typically occurs ~15 to 20 years after diagnosis (84–86). HD is caused by an expanded polyglutamine-coding CAG repeat on exon 1 of the *IT15* (*HTT*, Huntingtin) gene, located on chromosome 4 (87). This mutation is inherited in an autosomal dominant manner, therefore all patients have a 50% risk of passing the mutated gene on to each of their offspring (88). The hallmark neuropathological features of the disease are severe atrophy in the basal ganglia and cerebral cortex, and the presence of intranuclear aggregates throughout the brain (89).

A transgenic sheep model of Huntington's disease was developed to manifest the early, pre-symptomatic stages of the disease, to aid in studying the early molecular and neuropathological changes occurring in HD, and to test potential therapies (39). The HD sheep were developed by insertion of the full-length human *HTT* cDNA containing 73 CAG repeats into pronuclei, and subsequent implantation of the pronuclei into surrogate ewes. The resulting G0/5 line of transgenic sheep show robust expression of mutant *HTT* mRNA and protein throughout the brain (39, 90). Observations of the HD sheep in the field have reported no overt motor symptoms up to 5 years of age (91). General activity of the sheep measured using actimetry showed a clear circadian deficit in HD sheep at 18 months of age, which was exacerbated at 5 years of age (91). This disruption to sleep-wake cycles in HD sheep has been further characterized using EEG, and is postulated to be an early biomarker of disease in both sheep and human patients (92, 93). HD sheep brains show no volume changes or morphological differences up to 5 years of age, however do exhibit huntingtin-positive inclusions in several cortical regions from 18 months of age (90, 91). In addition, the transgenic sheep show some significant changes in neurochemical expression in the brain, as well as metabolic deficits in the cerebellum, liver, and plasma (90, 94–96).

Research efforts are currently focused on therapies for HD, particularly the use of gene silencing therapy to slow or halt disease progression. The HD transgenic sheep have undergone trials of gene silencing using RNA interference (RNAi). Artificial micro-RNA targeting exon 48 of the human *HTT* mRNA was able to reduce levels of mutant *HTT* mRNA and protein when injected unilaterally into the striatum (97). This study had the advantage of being able to assess targeting of the full-length gene, something which is lacking in most small animal models expressing only fragments of mutant *HTT* (m*HTT)*. However, the impact that the observed reduction in m*HTT* has on disease progression cannot be studied due to the lack of a robust clinical phenotype and only mild neuropathology in the transgenic sheep. This shortfall may be due to the nature of the model, that is, the transgenic sheep possess a mutated human *HTT* allele alongside their two intact ovine *HTT* alleles. Although this is useful for achieving the ultimate goal of targeting therapies specifically to the human gene, overt neuropathology and symptomology may be achieved by knocking in the mutation to the sheep genes.

#### Neuronal Ceroid Lipofuscinosis (CLN1 Batten Disease)

CLN1 Batten disease is a lysosomal storage disease with a similar disease phenotype and pathology as already described for CLN5 and CLN6 Batten disease. CLN1 disease is caused by mutations in the palmitoyl-protein thioesterase 1 (*PPT1*) gene, resulting in reduced enzymatic activity of PPT1 in the lysosome (45). Compared to other forms of NCL, CLN1 disease typically has a very early onset and rapid progression. Children with CLN1 disease will start to show developmental delay, motor decline, deterioration of speech, seizures, and loss of vision usually before 2 years of age (45, 98).

The most well studied animal model of CLN1 disease is a PPT1 null mouse, which has been useful for studying disease progression and trialing potential therapies (99, 100). To bridge the gap between small animal studies and translatable clinical research, Wishart and colleagues employed CRISPR-Cas9 technology to generate a sheep model of CLN1 disease, with the aim of recapitulating the complex disease pathogenesis in a larger animal (40). The CRISPR-Cas9 system was used to introduce the most common CLN1 disease-causing mutation into the sheep *PPT1* gene (40). Of 24 live births, genotyping revealed 3 animals had both alleles successfully edited. These PPT1 mutant animals showed progressive loss of vision and proprioceptive deficits, and a significantly reduced life span of just 17 months. Significant neuropathological abnormalities were observed post-mortem including a 30% reduction in brain mass, significantly enlarged cerebral ventricles, and thinning of cortical gray matter particularly in sensory regions (40). Although the end-stage disease pathology is compelling, it will be important to assess this model throughout the animals' lifetime to fully understand the degree to which it recapitulates the human disease. However, this work provides strong evidence that gene editing technologies can be utilized in sheep and may potentially remove the limitations previously imposed by the necessity to identify naturally occurring models of genetic diseases in sheep. Indeed, CRISPR-Cas9 technology is also being employed to create a model of CLN7 NCL in sheep *via* electroporation of *in vitro* derived embryos (101).

### Surgical Models

#### Traumatic Brain Injury

Animal models of traumatic brain injury (TBI) are useful for studying post-injury brain physiology and pathology and assessing efficacy of therapeutic interventions to improve outcomes. There are several different methods that can be employed to create an animal model of TBI, depending on the species and the research objectives. In the earliest models of TBI in sheep injury was induced using the fluid percussion (FPI) method, whereas more recently methods such as controlled cortical injury (CCI) and unconstrained impact acceleration have been utilized (102–105). FPI uses a pressure pulse system to deliver saline at high velocity to the surface of the cerebrum resulting in a focal injury. Using this method, Millen and colleagues were unable to elicit a physiological response to trauma in sheep, despite observing an acute hemodynamic and catecholamine response to the same methods in smaller animals such as cats and dogs (105).

Methods of direct brain deformation such as FPI and CCI require a craniotomy and often involve the head being constrained in a stereotaxic frame. These types of injuries do not therefore mimic the most common types of injuries in humans (i.e., those that result from collisions, blunt force trauma, or falls) with regards to head acceleration, deceleration, and rotation. The head impact model accounts for these components of the injury by creating impact on an unrestrained head. In addition, the head impact model does not require a craniotomy making it a less invasive procedure with less risk of surgical adverse events. Using the head impact model, researchers have elucidated the cascade of pathological events and the time course of changes in factors such as intracranial pressure, brain tissue oxygenation, and axonal injury following trauma (42, 102, 103, 106). A notable limitation to these impact models in sheep is the difference in the neuraxis, the axis of the brain and spinal cord, between sheep and humans. Being quadrupeds, sheep have an almost linear neuraxis, while it is curved at the cephalic flexure in humans. This should be considered when translating anatomical data related to rotational or acceleration injury in this model into a clinical context.

#### Stroke

**Figure 3:**
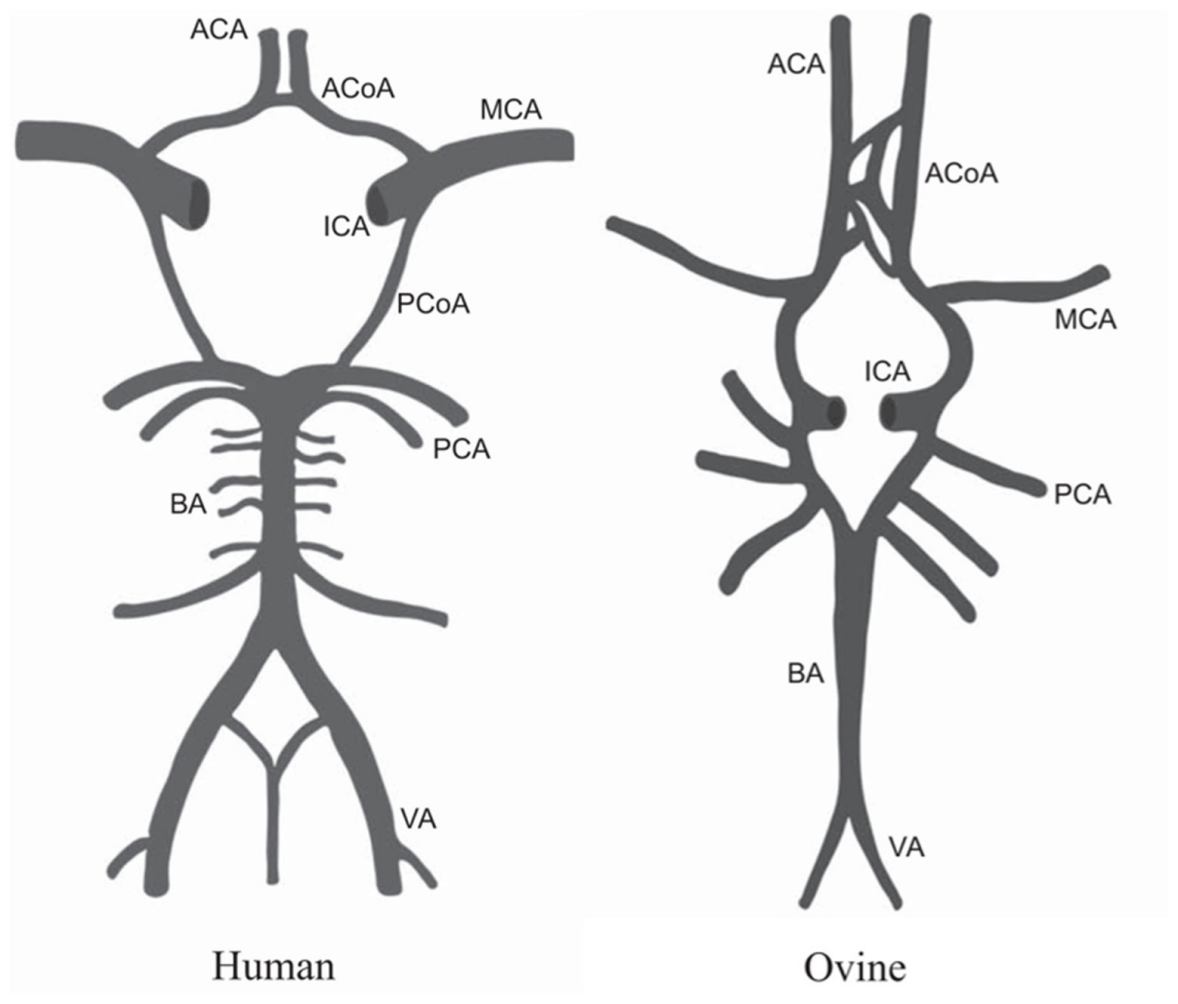
Comparison of the anatomy of the circle of Willis in humans and sheep. MCA, middle cerebral artery; ICA, internal carotid artery; ACoA, anterior communicating artery; ACA, anterior cerebral artery; PCoA, posterior communicating artery; PCA, posterior cerebral artery; BA, basilar artery; VA, vertebral artery. Figure adapted *from Large animal models of stroke and traumatic brain injury as translational tools* by Sorby-Adams et al. (15) with permission from The American Physiological Society.

Models of stroke were developed in large animals such as sheep to address shortcomings in small animal models and increase translatability of findings. In humans, the majority of ischemic strokes are a result of occlusion of the middle cerebral artery (MCA). The cerebral vasculature of sheep is largely the same as the human brain, with only minor differences in the architecture of the circle of Willis (Figure 3) observed in some samples studied, making sheep a valid model for studying cerebrovascular disease (107, 108).

Modeling ischemic stroke in sheep by MCA occlusion (MCAo) was first described by Boltze and colleagues in 2008 in an attempt to bridge the gap between successful therapies in small animals and failed clinical trials. One drawback in the use of sheep to model MCAo is the presence of the rete mirabile, a complex network of vessels within the cerebral vasculature of sheep that prevents intraluminal access to the MCA, meaning more invasive cranial surgery is required. Despite this, permanent occlusion of the MCA in sheep results in a robust, reproducible infarct characterized by deficits in cerebral blood flow and metabolic rate, cortical necrosis, neuroinflammation, and accompanying deficits in motor function (41). Temporary MCAo in sheep causes decreased oxygenation of brain tissue which returns to normal levels after reperfusion, resulting in less tissue swelling, neuronal cell death, and white matter injury, and an overall smaller infarct size in the long term (109, 110). Modeling acute ischemia in sheep has allowed researchers to develop clinically relevant imaging protocols to determine presence and extent of MCAo, and test early interventions such as therapeutic hypothermia (111, 112).

#### Spinal Cord Injury

The sheep spine and spinal cord have very similar anatomical features and dimensions to the human spine. The sheep spine consists of 7 cervical, 12-14 thoracic, and 6-7 lumbar vertebrae, with dimensions of the vertebral bodies, pedicles, spinous and transverse processes, and the spinal canal most similar to humans in the thoracic and lumbar regions (10, 11). Consequently, sheep have been used to model spinal cord injury, particularly contusive injuries, since the 1970's (113–115). These early studies were the first to characterize cell death, gliosis, axonal swelling, and vascular damage and noted the presence of irreversible damage as early as four hours after injury (113, 115).

More recently Wilson and colleagues employed the weight-drop method to create a moderate, incomplete spinal cord injury (SCI) in the thoracic section of the sheep spine, with the goal of developing a model to assess the therapeutic efficacy of spinal-cord stimulation (12). Sheep were trained to walk on a treadmill unassisted, which allowed the collection of a large number of gait metrics. One animal was implanted with an epidural stimulation array 5 months post-SCI and underwent gait analysis using a stimulation-on stimulation-off paradigm (12). There was a significant improvement in gait metrics, most notably in hind limb spasticity, during high intensity stimulation compared to baseline, stimulation-off, and lower intensity stimulation. One of the many advantages of using sheep to test therapeutic stimulatory electrode arrays is that the devices are directly translatable to human patients with regard to array size and placement, owing to the similarities in anatomy between the sheep and human spine and spinal cord. In addition, sheep are being utilized to test efficacy of epidural and intradural devices being developed with the aim of improving treatments for SCI and neuropathic pain (116–118).

#### Epilepsy

Seizure activity can be induced in animals *in vivo* by intracortical administration of penicillin which has an inhibitory effect on the neurotransmitter ɤ-aminobutyric acid (GABA), therefore disrupting neurotransmission and normal brain activity. Opdam and colleagues were the first to show that penicillin administration to the frontal cortex of sheep could elicit focal seizures with secondary spread, accompanied by behavioral seizure activity (119). Using sheep to model epilepsy in this way has allowed for concurrent EEG and functional MRI data collection, to deepen understanding of seizure generation and spread, and blood flow and oxygenation levels in relation to EEG measures.

Deep brain stimulation (DBS) has been suggested as a potential treatment for drug-resistant epilepsy, with many of the trials of devices, neuroanatomical targets, and stimulation patterns being conducted in sheep models. MRI-guided placement of a DBS device and recording electrodes in the sheep brain provided a proof-of-concept that high-frequency DBS of the thalamus was able to inhibit both induced and spontaneous seizure activity, even beyond the period of stimulation (43). Further to this, sheep were used to test a new clinical-grade DBS device that was able to stimulate and record neural responses simultaneously. The device was implanted into either the anterior thalamic nucleus or hippocampus and was able to chronically stimulate and record neural activity in free roaming animals for over a year (120). Trials such as these in the sheep brain have produced clinically relevant and translatable results.

Sheep models of epilepsy have also been an invaluable tool in studying the possible causes of sudden unexplained death in epilepsy (SUDEP). Monitoring of EEG, ECG, airway flow, arterial pressure and blood gases during induced seizures in sheep identified increased pulmonary vascular pressure as the key parameter that led to death following seizures (121, 122). In addition, a concurrent fall in oxygen partial pressure and rise in carbon dioxide partial pressure following seizure was noted in animals that subsequently died. Due to the fact that airway flow was monitored *via* tracheostomy in these studies, death was attributed to central apnea as opposed to obstructive apnea (121, 122). Post-seizure central apnea has since been characterized in populations of epilepsy patients and been postulated as a biomarker for risk of SUDEP (123).

## Limitations of Sheep

Sheep do have some limitations in their use as a model species including the availability of ovine specific reagents for (histo)pathological studies and the practicalities associated with developing and maintaining a research flock. Sheep require more specialized large animal housing, surgical facilities and husbandry which are not commonplace in many animal research facilities. Sheep are less inbred than small research animals such as rodents, and genetically manipulated or naturally occurring models are not so readily accessible to the wider research community. In addition, sheep have long gestations (4.7–5 months) and lifespans and are seasonal breeders producing 1–3 offspring annually therefore developing research flocks and compiling data from them takes longer compared to small animals, which can put strains on research funding and publication, and means important contributions to the field take more time. Additionally, because ovine disease models are less well established than rodents, there are fewer validated sheep-specific neurological and cognitive tests available. Availability of reagents specialized for use on sheep tissue, particularly antibodies, is also limited, although this is improving as studies on sheep have become more common in recent years (60, 124). In addition, because the homology between sheep and human genomes is relatively high (>85%), often antibodies developed for use on human tissue will also bind to sheep tissue.

With regard to modeling neurological conditions specifically, there are some differences in anatomy between sheep and humans that can limit the uses of the model. Being quadrupeds, sheep have a different neuraxis than humans which can impact the development and assessment of models of TBI and SCI. One of the most important neuroanatomical differences is the presence of the rete mirabile in sheep making development of ovine models of stroke more complex, as discussed in the above section on Stroke.

## Future Potential for Sheep

As our understanding of the anatomy and physiology of the nervous system in sheep continues to grow, their potential uses in biomedical research expand. The potential to model neurodegenerative diseases such as Alzheimer's disease (AD) and Parkinson's disease (PD) in sheep has been explored although to our knowledge no ovine models of this nature are currently in active study. Inducing PD in sheep was first trialed in the 1980's by administration of 1-methyl-4-phenyl-1,2,3,6-tetrahydropyridine (MPTP), a neurotoxin that is commonly used to create PD models due to its targeting of dopaminergic neurons. These early studies showed that MPTP delivered intravenously to sheep was able to induce permanent motor deficits and neuropathology reminiscent of PD (125–127). Tau neurofibrillary tangles, a hallmark of AD pathology, were first observed in the aged sheep brain in the 1990's (128, 129). More recently, it has been shown that there is a high degree of homology between the human and sheep amino-acid sequences of AD-associated proteins such as amyloid precursor protein (APP). In addition, the fragments of APP found in aged sheep were the same as those commonly found in humans (130). Amyloid-β has been detected in the CSF, as well as plaques in the cerebral cortex and hippocampus, of aged sheep (130). These studies show that sheep have the correct genetics and cellular processes in place to exhibit AD neuropathology and therefore may make a useful model of the disease in future. As opposed to spending time and resources on sheep husbandry and waiting for them to develop this pathology naturally, it has been suggested that use of genetic manipulation to induce disease could be used to develop a model (130).

Healthy control sheep are also useful for testing safety and mechanisms of action of potential therapeutic drugs or devices, and to refine neurosurgical techniques. For example, sheep have been used to validate a method of blood brain barrier (BBB) opening to allow more effective delivery of drugs to the brain (131). This ultrasound-mediated BBB opening method had previously been tested in rodents, however sheep represented a more relevant model due to its similar skull composition and thickness to humans.

## Conclusions

Studies in animal models of neurological disorders are essential not only for developing an insight into the underlying neurobiology, but also for assessing safety and efficacy of potential therapeutic drugs or interventions. Large animal models are of particular importance with regards to pre-clinical studies, as findings from large animals are more relevant to the human central nervous system. Sheep are excellent candidates for modeling neurological disorders and have already demonstrated their validity with several pre-clinical trials in sheep leading to the initiation of human clinical trials. In addition, scientists utilizing sheep have shown great ingenuity in both experimental induction of neurological conditions and in developing or adapting *in vivo* methodologies to study sheep anatomy, physiology, and behavior. With substantial progress in genetic screening and gene editing technologies, the potential for modeling diseases in sheep will only continue to increase.

## Author Contributions

SM: conceptualization, writing—original draft preparation, and review and editing. NM: writing—review and editing. Both authors contributed to the article and approved the submitted version.

## Conflict of Interest

The authors declare that the research was conducted in the absence of any commercial or financial relationships that could be construed as a potential conflict of interest.

## Publisher's Note

All claims expressed in this article are solely those of the authors and do not necessarily represent those of their affiliated organizations, or those of the publisher, the editors and the reviewers. Any product that may be evaluated in this article, or claim that may be made by its manufacturer, is not guaranteed or endorsed by the publisher.
